# Insight Into the Formation Paths of Methyl Bromide From Syringic Acid in Aqueous Bromide Solutions Under Simulated Sunlight Irradiation

**DOI:** 10.3390/ijerph17062081

**Published:** 2020-03-20

**Authors:** Hui Liu, Tong Tong, Yingying Pu, Bing Sun, Xiaomei Zhu, Zhiyu Yan

**Affiliations:** College of Environmental Science and Engineering, Dalian Maritime University, Dalian 116026, China; tongtong@dlmu.edu.cn (T.T.); py1120181596@dlmu.edu.cn (Y.P.); sunb88@dlmu.edu.cn (B.S.); zhuxm@dlmu.edu.cn (X.Z.); yanzy@dlmu.edu.cn (Z.Y.)

**Keywords:** methyl bromide, syringic acid, photochemical production, reaction paths

## Abstract

Methyl bromide (CH_3_Br) is one of the largest natural sources of bromine in the stratosphere, where it leads to ozone depletion. This paper reported the photochemical production of CH_3_Br from syringic acid (SA) that has been used as an environmentally relevant model compound for terrestrially-derived dissolved organic matter. The formation of CH_3_Br increased with the increase of bromide ion concentration ranging from 0.8 to 80 mmol L^−1^. Ferric ions (Fe(III)) enhanced CH_3_Br production, while chloride inhibited it, with or without Fe(III). Meanwhile, methyl chloride (CH_3_Cl) was generated in the presence of chloride and was inhibited by Fe(III). The different effects of Fe(III) on the formation of CH_3_Cl and CH_3_Br indicate their diverse formation paths. Based on the intermediates identified by liquid chromatography-mass spectrometry and the confirmation of the formation of Fe(III)-SA complexes, it was proposed that there were two formation paths of CH_3_Br from SA in the bromide-enriched water under simulated sunlight irradiation. One path was via nucleophilic attack of Br^−^ on the excited state protonation of SA; the other was via the combination of methyl radical and bromine radical when Fe(III) was present. This work suggests that the photochemical formation of CH_3_Br may act as a potential natural source of CH_3_Br in the bromide-enriched environmental matrix, and helps in better understanding the formation mechanism of CH_3_Br.

## 1. Introduction

Methyl bromide (CH_3_Br) is the most abundant brominated gas in the troposphere, which is transported to the stratosphere, releasing bromine atoms, which then destroy ozone catalytically. The global averaged mixing ratio of CH_3_Br is 7.9 ppt (parts per trillion), representing >50% of total organic bromine in the troposphere [1]. Although CH_3_Br has lower atmospheric abundance compared with methyl chloride (CH_3_Cl) at 545 ppt, release of a Br atom is more destructive to stratospheric ozone than a Cl atom [2]. In the past 20 years, a lot of work has been done to quantify the sources and the sinks of CH_3_Br. However, large uncertainties in its budgets still remain. Estimates of global sinks of CH_3_Br (148 Gg yr^−1^) are significantly unbalanced by ~30% with the estimates of global sources (112 Gg yr^−1^) [3]. It is estimated that methyl halides originate in large part from natural sources which are believed to account for ~60–80% of the global CH_3_Br [3]. These natural sources include oceans, biomass burning (which can also be anthropogenic), wetlands, Brassica crops, and fungus [3,4,5,6,7]. The ocean, as one of the major sources and also major sinks for CH_3_Br, plays an important role in its atmospheric budget [8,9]. While the open ocean is a net sink for CH_3_Br, the coastal ocean often exhibits significant super saturation in previous studies [10]. For example, investigation of the distributions of halocarbons in the marine boundary of air and surface seawater indicated that the Yangzi River Estuary and adjacent coastal area were net sources of atmospheric CH_3_Br [11]. Therefore, more attention should be paid to the process of CH_3_Br production in the estuarial and coastal ocean areas. 

In principle, natural processes for CH_3_Br production proceed through an assortment of biological [12] and/or abiotic pathways. Focusing on the abiotic pathways, one abiotic production pathway of CH_3_Br was suggested for a methoxy moiety (-OCH_3_) reacting with halides directly via a nucleophilic displacement reaction [13,14]. In addition, CH_3_Br production from biomass burning was thought to proceed in a path via methanol production during pyrolysis [15]. In both of these proposed pathways, lignin and pectin components which can provide a large amount of methoxy moieties seem essential for CH_3_Br production. Keppler et al. found that CH_3_Br was generated abiotically through alkylation of Br ions during the oxidation of soil organic matter by ferric iron (Fe^3+^) [16]. Moore reported that some lignin model compounds could act as the carbon precursor to form CH_3_Cl in saline waters [17]. Considering that humic acid is partially originated from terrestrial plants containing large amounts of lignin [18], it is reasonable to speculate that humic acid could act as the precursor of methyl halides. 

Humic acid deriving from terrestrial biota contains a large assemblage of complex chemical structures of polyphenol, carboxyl, methoxyl, and quinone functionalities, and is distributed as a component of dissolved organic matter (DOM) in estuary and coastal ocean areas [19,20]. As we know, DOM can absorb sunlight energy and plays an important role in the photochemical transformation of organic contaminants through producing an excited triplet state, reactive oxygen species, and reactive halogen species, and so on [19,21,22]. In estuarine waters, terrestrial DOM and high levels of halides, e.g., chloride and bromide, occur simultaneously [23], providing a suitable situation for the production of organo-halogens [24]. There are a number of reports on the photochemical production of methyl halides (chiefly methyl iodide (CH_3_I) and CH_3_Cl) from DOM in seawater [17,25,26]. The photochemical production of CH_3_I from DOM which has been demonstrated to be a major source of CH_3_I in marine environments involved the release of methyl radicals, ^•^CH_3_ from DOM, and their combination with iodine radicals, I^•^ [25,27,28]. In contrast, the photochemical production of CH_3_Cl was thought to proceed through a methoxy moiety of DOM directly reacting with chloride ions via a nucleophilic displacement reaction [17,29,30]. Thus it can be seen that the productions of CH_3_Cl and CH_3_I are proposed to occur via two different pathways. However, the photochemical generation of CH_3_Br from DOM is less understood until now. 

The present work attempted to investigate the photochemical production of CH_3_Br from DOM and to gain insight into the path of CH_3_Br generation. Considering that structural moieties on syringic acid (SA) have been identified in terrestrially derived DOM [17], and Benner and Opsahl have shown that aryl-methoxy groups on syringyl moieties of lignin are photochemically labile and preferentially degraded as terrigenous carbon moves from freshwater to the open ocean [31], SA was used as a model compound for DOM in the experiment. This study first confirmed the formation of CH_3_Br from SA in the aqueous bromide solutions, then, by comparing the different generation profiles of CH_3_Br and CH_3_Cl as a function of ferric ions concentration, proposed two reaction pathways for the photo-initiated formation of CH_3_Br described. 

## 2. Materials and Methods

### 2.1. Reagents

Syringic acid (4-hydroxy-3,5-dimethoxybenzoic acid) was purchased from Molekula Ltd., United Kingdom. Liquid standards of CH_3_Br and CH_3_Cl (200 μg mL^−1^ in methanol) were purchased from Accustandard, USA. Sodium bromide (NaBr), sodium chloride (NaCl), FeCl_3_·6H_2_O and other chemicals were reagent grade. Ultrapure water (18 MW cm) was obtained with a Millipore water purification water unit to prepare all aqueous solutions. 

### 2.2. Irradiation Experiments

The irradiation experiments were performed in a solar simulator (Phchem III, Beijing Newbit Technology Co., Ltd, China) equipped with a 500 W xenon arc lamp and filters to cut off light with a wavelength below 290 nm. Considering the attenuation of light in a water body, the average light intensity in the euphotic zone should be much lower than the surface water (around 85 mW cm^−2^). Thus, the light intensity of the solar simulator was set at 15 mW cm^−2^. The reactor was a round bottom sealed quartz tube (3.5 cm o.d.; volume ca. 125 mL) with one outlet (4 mm o.d.) in the middle of the bottom. Details are in Text S1, Figure S1, and Figure S2 in the supplementary information. The dark control tubes wrapped in Al foil were also placed in the solar simulator. Two parallel samples were set up in each experiment. 

### 2.3. Analysis Methods 

The concentrations of CH_3_Br were analyzed by gas chromatography-mass spectrometry (GC-MS, Aglient 7890B/5977C, Santa Clara, CA, USA) equipped with a purge-and-trap sample concentrator (Eclipse 4760, College Station, TX, USA). Briefly, aqueous samples were injected into a 25 mL-purge tube, subsequently purged with ultrapure nitrogen at 40 mL min^−1^ for 11 min. The extracted gases were pre-concentrated in the trap tube containing VOCARB 3000, and then released from the trap column by heating to 240 °C, finally were introduced into GC-MS. A DB-VRX capillary column (60 m × 250 μm × 1.4 μm, Agilent Technologies, Palo Alto, CA, USA) was used. The inlet worked in a split model with a split ratio of 20:1 and the temperature was set at 150 °C. The oven temperature was initially set at 32 °C for 6 min and rose to 180 °C at 20 °C min^−1^ then held at 180 °C for 4 min. The select ion monitor (SIM) mode was used for quantitative analysis of CH_3_Br with m/z of 94 and 96, and CH_3_Cl with m/z of 50 and 52. The detection limits (signal to noise ratio, S/N = 3) of CH_3_Cl and CH_3_Br were 62 and 4.1 pmol L^−1^, respectively, and the relative standard deviations of replicate analyses (n = 6) were within 6% and 8%, respectively. 

The photolysis intermediates of SA were characterized by triple quadrupole liquid chromatography-mass spectrometry (LC-MS). After irradiation, in order to maximize the recovery of the acid products, aqueous samples were acidized to pH2.0 using sulfuric acid, then passed through SPE cartridges at a flow rate of 1~2 mL min^−1^ after the cartridges were activated with 3 mL methanol and 3 mL Milli-Q water in sequence. Then the intermediates were eluted by methanol. The LC-MS system consisted of an Agilent QQQ 6410B MS system equipped with electro spray ionization (ESI) interface and an Agilent 1200SL system (Agilent Technologies, Santa Clara CA, USA). The analytical column was XTERRA® MS C18 (2.1 × 100 mm, 3.5 μm, Waters, Milford, MA, USA). Mobile phases A and B were water with 0.1% HCOOH and acetonitrile, respectively, using a gradient from 10% B at 0.1 min to 60% B at 10 min, which was kept isocratic for 2 min, followed by a gradient back to the initial 10% B at 20 min with a flow rate of 0.25 mL min^−1^. The injection volume was 10 μL. The source temperature of the heated capillary was set at 350 °C, and the source voltage was 4.0 kV. 

The solutions of Fe(III)–SA complex were stirred for 1 h in the dark to reach equilibrium. Their Ultraviolet –visible (UV–Vis) absorption spectra were measured using a spectrophotometer (Hitachi UH5300, Ibaraki, Japan). 

## 3. Results and Discussion

### 3.1. Formation of CH_3_Br from SA in Aqueous Bromide Solutions

**Figure 1:**
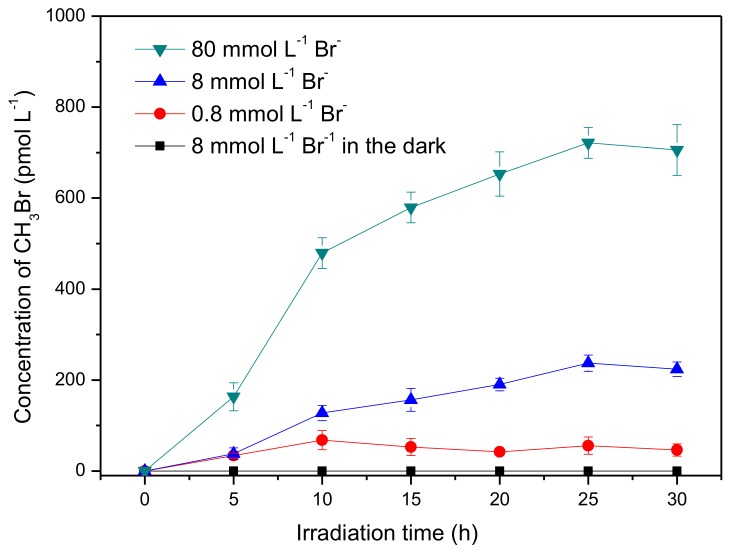
Formation of methyl bromide (CH_3_Br) in aqueous solutions containing syringic acid (50 μmol L^−1^) and bromide ions (0.8~80 mmol L^−1^). Error bars represent one standard deviation.

The formation of CH_3_Br was first investigated in the presence of 50 μmmol L^−1^ SA and 8 mmol L^−1^ bromide ions under simulated sun-light irradiation. As shown in Figure 1, about 220 pmol L^−1^ CH_3_Br was generated after irradiation of 30 h, whereas no detectable CH_3_Br was formed in the dark. This result indicates that CH_3_Br was produced through a photochemical reaction of SA and bromide, which is in agreement with the previous reports of the formation of CH_3_Cl by Moore and Dallin et al. [17,29]. In addition, Moore inferred the production of CH_3_Cl was not from SA directly but an intermediate of SA photolysis, somewhat a quinone derivative [17]. Although the loss of SA was almost negligible during irradiation (Figure S3), CH_3_Br formation was lowered after long-term irradiation. Here, the slow accumulation of CH_3_Br is speculated to be related to the slow formation rate of the active intermediate of SA and/or the degradation of CH_3_Br itself. 

Bromide ions in seawater are at an average concentration of 0.8 mmol L^−1^, and are further enriched in the nanolayer close to the air–sea interface; they can even reach a level of dozens of mmol L^−1^ at the marine boundary layer [32,33]. Consequently, the effect of bromide ions on the formation of CH_3_Br was investigated with concentrations ranging from 0.8 mmol L^−1^ to 80 mmol L^−1^. It was found that CH_3_Br production increased with increasing bromide concentration. The formation rates of CH_3_Br at the first 10 h in the presence of 0.8, 8.0 and 80 mmol L^−1^ Br^−^ were estimated to be 6.81, 12.7, and 47.9 pmol L^−1^h^−1^, respectively. This result indicates that Br^−^ acted as the limiting factor for the yield of CH_3_Br, which is consistent with the geochemical behavior of bromide in soil that bromide was the limiting factor for the bromination of soil organic matter [34]. As described above, the bromide concentration is further enhanced in the thin film of the surface seawater; it could be proposed that the emission of CH_3_Br might be more significant at the air–sea interface [32,33], although, of course, various other anions and cations in seawater may have different impacts on this process as well. 

### 3.2. Effect of Chloride on the Formation of CH_3_Br

**Figure 2:**
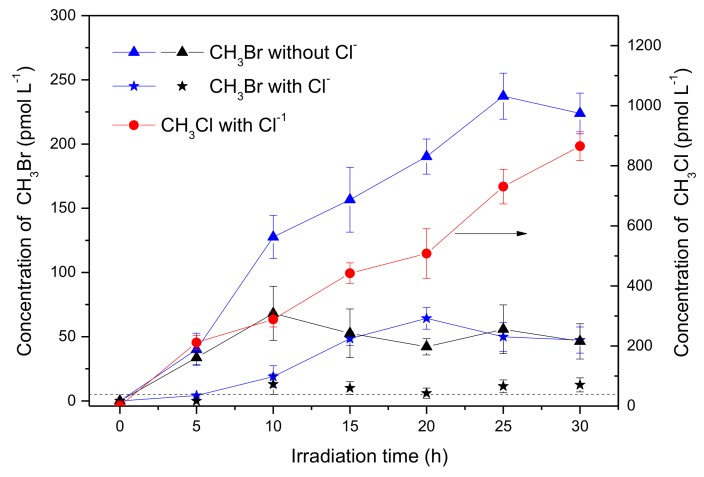
Formation of CH_3_Br (triangle and star) and methyl chloride (CH_3_Cl; circle) in the solutions containing 50 μmol L^−1^ SA, 8 mmol L ^−1^ Br^−^ (blue) and 0.8 mmol L^−1^ Br^−^ (black) in the presence or in the absence of 0.5 mol L^−1^ Cl^−^. There was no obvious difference for CH_3_Cl in the presence of 0.8 or 8 mmol L^−1^ Br^−^. Dashed line indicates the detection limit of CH_3_Br. Error bars represent one standard deviation.

The experiment was then carried out with the addition of 0.5 mol L^−1^ NaCl, in order to know the effects of chloride on the production of CH_3_Br. When [Br^−^] was 8 mmol L^−1^, the concentration of CH_3_Br in the presence of Cl^−^ after 30 h of irradiation reduced to 47.3 pmol L^−1^, meaning about 20% of the sample was without Cl^−^ (Figure 2, blue curves), showing that Cl^−^ inhibited the production of CH_3_Br. Meanwhile, a significant amount of CH_3_Cl was generated, which reached 865 pmol L^−1^ after 30 h of irradiation (Figure 2, red curve). When [Br^−^] was 0.8 mmol L^−1^, (the ratio of Cl: Br was 625 close to the natural seawater), the circumstance were similar, where CH_3_Br decreased distinctly (Figure 2, black curves). These results indicate that chloride was a forceful competitor for bromide to react with SA forming CH_3_X (X = Cl, Br), and also suggest that CH_3_Br might be generated through a similar pathway to CH_3_Cl under the experimental conditions. 

**Figure 3:**
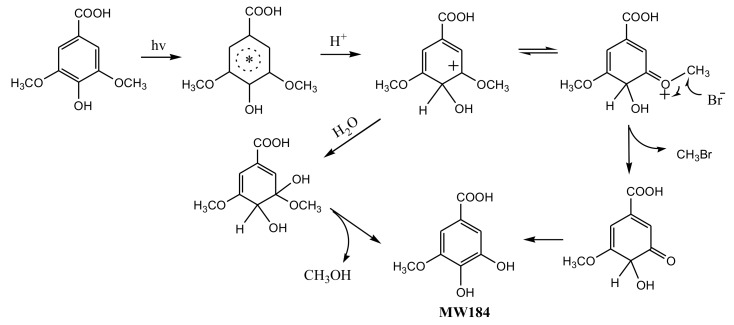
A possible formation pathway of CH_3_Br from syringic acid (SA) under irradiation.

To understand the photochemical transformation pathway of SA and the formation mechanism of CH_3_Br, LC-MS was employed to identify the intermediates of this reaction (Figure S4). The intermediate with molecule weight (MW) 184 confirmed using ESI(−) MS was attributed to 3-methoxy-4,5-dihydroxybenzoic acid, a demethylation product of SA. Hence, it could be assumed that the methyl group of CH_3_Br originated from the methoxy group of SA. This proposal was consistent with the formation pathway of CH_3_Cl from SA which has been well demonstrated by Dallin and Moore et al. [17,29]. The reaction proceeds via aromatic ring protonation followed by demethoxylation, as shown in Figure 3. The first step of CH_3_Br formation is protonation, i.e., SA is initiated by the protonation of excited state benzene ring (ipso positions with OCH_3_). The arenium ion intermediate has a minor resonance contributor with the positive charge distributed onto the methoxy oxygen. The second step is demethylation, involving nucleophilic attack of Br^−^ onto the methoxy carbon resulting in C_methyl_–O cleavage [29]. When chloride and bromide ions coexist, attacking the methoxy carbon yields both CH_3_Cl and CH_3_Br. Since 0.5 mol L^−1^ chloride ions was more competitive for this process than 0.8 and 8 mmol L^−1^ bromide, the production of CH_3_Br decreased. 

### 3.3. Formation of CH_3_Br and CH_3_Cl in the Presence of Ferric Ions

**Figure 4:**
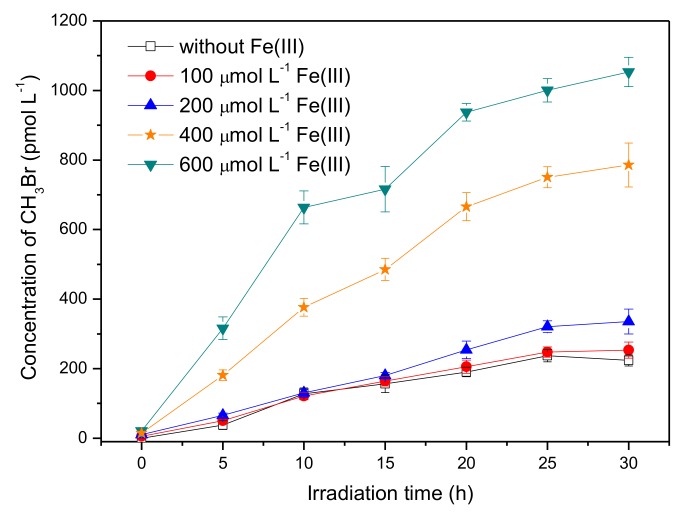
Effect of Fe(III) on the formation of CH_3_Br from 50 μmol L^−1^ SA with 8 mmol L^−1^ NaBr. Error bars represent one standard deviation.

Iron is a universal element in the natural aqueous environment and plays an important role in the photochemical transformation of pollutants [35]. Here, the experiments were carried out by adding ferric ions, Fe(III), with concentrations ranging from 0 to 600 μmol L^−1^, into the solution containing 50 μmol L^−1^ SA and 8 mmol L^−1^ Br^−^. The pH of SA solutions with 0, 100, 200, 400, and 600 μmol L^−1^ Fe(III) was 5.3, 3.9, 3.5, 3.2, and 3.0, respectively. Figure 4 shows that Fe(III) had a significant promotion effect on the formation of CH_3_Br. The concentration of CH_3_Br in the presence of 600 μmol L^−1^ Fe(III) reached around 930 pmol L^−1^ after 20 h of irradiation, which was about four times higher than that without Fe(III).

In general, Fe(III) in the solution exists as iron-hydroxyl complexes including Fe(OH)^2+^, Fe(OH)_2_^+^, Fe_2_(OH)_2_^4+^, etc. Among them, Fe(OH)^2+^ is the most photochemical active species that has been identified to produce ^•^OH when irradiated (Equation (1)) [35]. As we know, ^•^OH is a strong oxidant and can oxidize X^−^ to produce reactive radical species, X^•^/ X_2_^•−^, where X=Br, Cl (Equations (2)–(4)) [36,37].

Fe(OH)^2+^ + *hν→* Fe^2+^ + ^•^OH (1)

^•^OH + X^−^ ⇄ HXO^•−^ (2)

HXO^•·−^ + H^+^ ⇄ X^•^ + H_2_O (3)

X^•^+ X^−^ →X_2_^•−^ (4)

**Figure 5:**
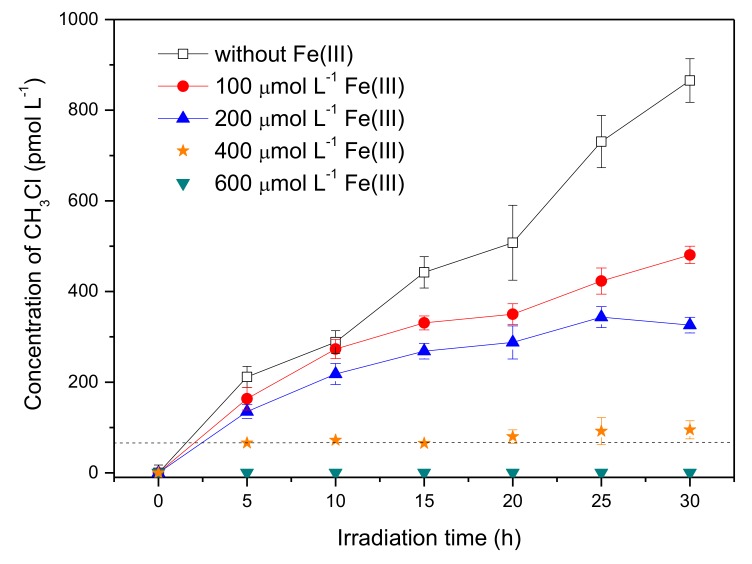
Effect of Fe(III) on the formation of CH_3_Cl from 50 μmol L^−1^ SA with 0.5 mol L^−1^ NaCl. Dashed line indicates the detection limit of CH_3_Cl. Error bars represent one standard deviation.

Considering the presence of reactive radical species in the systems containing Fe(III), bromine radical species were proposed to be the active intermediate for the production of CH_3_Br, which was a different formation pathway from CH_3_Cl. Previous studies on the formation of CH_3_Cl have demonstrated that CH_3_Cl generation occurred via a nucleophilic substitution by chloride ions, and the reactive radical species had no effect on CH_3_Cl formation [17,30]. In order to figure out the distinction between the generation of CH_3_Br and CH_3_Cl, the concentration of CH_3_Cl in the presence of Fe(III) was monitored as well. The result is displayed in Figure 5. It is notable that the formation profile of CH_3_Cl was quite different from CH_3_Br; that Fe(III) reduced the formation of CH_3_Cl sharply. The concentration of CH_3_Cl upon 20 h of irradiation decreased from 507 pmol L^−1^ to undetectable with Fe(III) increasing from 0 to 600 μmol L^−1^. The disparate effects of Fe(III) on the formation of CH_3_Cl and CH_3_Br suggests their different formation pathways in the presence of Fe(III). 

**Figure 6:**
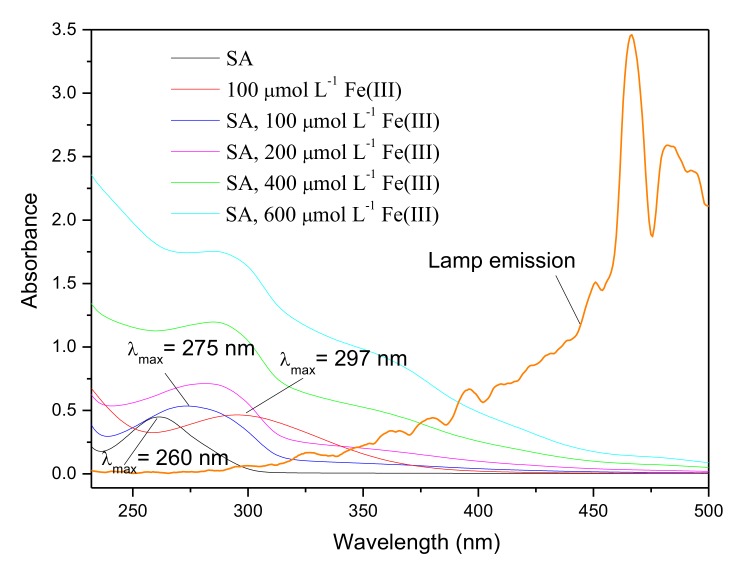
UV–Vis spectra of 50 μmol L^−1^ SA, Fe(III) and Fe(III)-SA complexes with different concentration of Fe(III), and the emission spectrum of the Xenon lamp.

Fe(III) is liable to be chelated by high affinity carboxylates, such as citrate and oxalate, to form Fe(III)-ligand complexes that exhibit appreciable reactivity to give out reactive species, such as ^•^OH, and ferrous ions, Fe(II), upon irradiation [35,38]. SA contains -COOH and -OH groups, and can act as a ligand to complex with Fe(III), and hence it promotes the formation of reactive species. The UV–Vis absorption spectra of SA with different concentration of Fe(III) are shown in Figure 6. SA exhibited a peak at 260 nm whereas the spectrum of Fe(III) showed a peak around 300 nm. When 100 μmol L^−1^ Fe(III) interacted with SA, the absorption profile exhibited a strong peak at 275 nm. This red shifted spectrum (large differences greater than 15 nm among peaks) was clearly distinguishable. According to Singh and Kumar’s study on the complex formation of Fe(III) and SA, the red shift of the spectrum provided the evidence that chelation of Fe(III) took place where SA acted as a ligand, forming Fe(III)-SA complex [39]. Fe(III)-ligand complexes can undergo ligand-to-metal charge transfer (LMCT) upon irradiation and consequently result in the enhancement of ^•^OH generation [35,38]. Considering the promotion effect of Fe(III) on the production of CH_3_Br, it is speculated that Fe(III)-SA and the corresponding ^•^OH are essential for the generation of CH_3_Br. 

**Figure 7:**
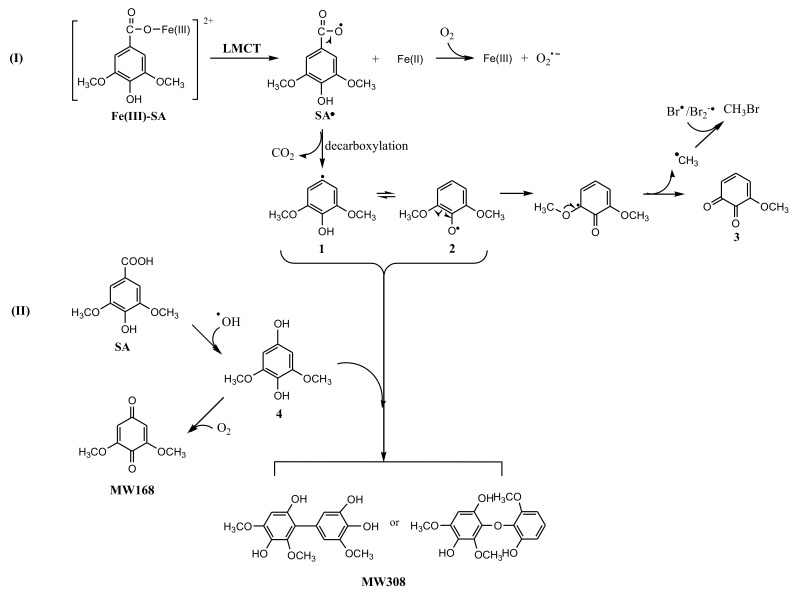
A possible formation pathway of CH_3_Br in the Fe(III)-SA system.

The reaction products in Fe(III)-SA system were analyzed using LC-MS. Besides 3-methoxy-4,5-dihydroxybenzoic acid, two new intermediates were detected. One product with MW 168 identified by ESI(+) MS was attributed to be 3,5-dimethoxy-1,4-benzoquinone, and the other one with MW 308 was attributed to be the dimer product (Figure S5 and S6). The proposed reaction pathway is displayed in Figure 7. Route I, an electron transferring from SA to Fe(III) in Fe(III)-SA complex resulted in Fe(II) and SA radical, SA^•^, with unpaired electron distributed on the carboxyl oxygen. Then SA^•^ released CO_2_ through decarboxylation reaction forming 2,6-dimethoxyl-phenol radical with the unpaired electron distributed on the aromatic carbon (**1**), or its resonance contributor with the unpaired electron distributed onto the hydroxyl oxygen, i.e., 2,6-dimethoxyl-phenoxy radical (**2**). The cleavage of C_methoxyl_–O resulted in methoxy-quinone (**3**) and methyl radical, ^•^CH_3_, which then recombined with Br^•^ to produce CH_3_Br. As the formation of CH_3_ radical was a minor reaction, the methoxy-quinone was not detectable by LC-MS. Route II, another reaction path, was dominated by ^•^OH where SA reacted with ^•^OH, forming 2,6-dimethoxyl-1,4-hydroquinone (**4**), which was then oxidized by oxygen forming 3,5-dimethoxy-1,4-benzoquinone (MW168). The product with MW308 might be generated by the combination of **4** with **1** or **2**. 

From the results of the above analysis, it could be hypothesized that CH_3_Br was primarily formed through a radical combination pathway in the presence of Fe(III). However, it was not able to be ruled out that the nucleophilic substitution path as shown in Figure 3 still existed, but played a minor role. Based on this proposal, it was not difficult to understand why Fe(III) enhanced the production of CH_3_Br. On the one hand, Fe(III) led to the generation of ^•^OH that can oxidize Br^−^ to produce reactive bromine radical species (Equations (2)–(4)); on the other hand, the LMCT of Fe(III)-SA resulted in the formation of methyl radical. That is, Fe(III) enhanced the formation of CH_3_Br by providing both ^•^Br and ^•^CH_3_ moieties. However, CH_3_Cl formation decreased sharply after adding Fe(III). The reason for this is that CH_3_Cl is generated through the nucleophilic substitution of Cl^−^ where protonation of excited-state benzene ring plays an important role [29,30]. The addition of Fe(III) lowering the solution pH could weaken the protonation of the excited state SA, and consequently reduce the formation of CH_3_Cl [29,40]. 

One more thing that should be examined is why CH_3_Cl was not produced through the radical combination pathway? This can be explained from the perspective of the formation rate of chlorine radicals from ^•^OH. Actually, Cl^−^ is an ineffective ^•^OH scavenger because the intermediate HClO^•−^ primarily reverts to ^•^OH and Cl^−^ (Equation (2)). For example, the rate constant for Equation (2) where X = Cl, *k_+_* (forward) is 4.0 × 10^9^ M^−1^s^−1^, and *k_−_* (backward) is 6.0 × 10^9^ M^−1^s^−1^; while X = Br, the *k*_+_ is 1.1 × 10^10^ M^−1^s^−1^, and k_−_ is 3.3 × 10^7^ M^−1^s^−1^ [37]. Consequently, about >99.98% HClO^•−^ will reform ^•^OH and Cl^−^, and will not form chlorine radical; in contrast, only 24% of HBrO^•−^ will reform ^•^OH and Br^−^. Therefore, Br^•^ has an order of magnitude dominance over Cl^•^. In addition, according to the calculation results of Parker et al., Cl^•^ is only present at a higher concentration relative to ^•^OH in more acidic solutions with pH lower than 3.0 [37]. Furthermore, Cl^•^ is more reactive than Br^•^, and can react with H_2_O more easily (backward reaction of Equation (3)). For example, *k* for Cl^•^ and H_2_O is 1.3 × 10^3^ M^−1^s^−1^, while the *k* for Br^•^ and H_2_O is 1.36 M^−1^s^−1^ [37]. Therefore, chlorine radical was hardly generated under the experimental conditions. 

**Figure 8:**
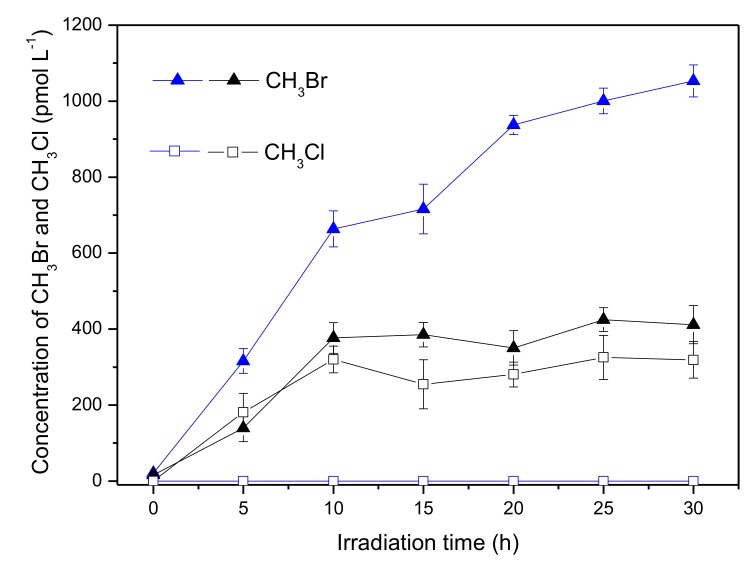
Formation of CH_3_Br (triangle) and CH_3_Cl (square) in the solutions containing 50 μmol L^−1^ SA, 600 μmol L^−1^ Fe(III) and 8 mmol L^−1^ Br^−^ in the presence (black) and in the absence (blue) of 0.5 mol L^−1^ Cl^−^. Error bars represent one standard deviation.

The effect of chloride on the formation of CH_3_Br and CH_3_Cl in the presence of Fe(III) is demonstrated in Figure 8. For CH_3_Br, chloride decreased its formation nearly from 1000 to 400 pmol L^−1^, which was similar as that in the absence of Fe(III) (Figure 2). Meanwhile, there was formation of CH_3_Cl in the presence of chloride. It is noteworthy that no detectable CH_3_Cl was generated in the coexistence of 600 μmol L^−1^ Fe(III) and Cl^−^ (Figure 5); however, CH_3_Cl was generated with a concentration around 300 pmol L^−1^ in the coexistence of Fe(III), Br^−^ and Cl^−^ (Figure 8, black square). This is probably due to the existence of the different halogen radicals in the reaction systems containing Fe(III), Br^−^ and Cl^−^. In fact, the mixed-halogen radical (BrCl^•−^) should be the major halogen radical that could be formed through the following Equations. [37]. The unit of the rate constant is M^−1^s^−1^.

Cl^•^ + Br^−^ ⇄ BrCl^•−^*k_+_* = 1.2 × 10^10^, *k_−_* = 1.9 × 10^3^ (5)

Br^•^ + Cl^−^ ⇄ BrCl^•−^*k_+_* = 2.3 × 10^8^, *k_−_* = 6.1 × 10^4^ (6)

HClO^•−^ + Br^−^ ⇄ BrCl^•−^ + OH^−^*k_+_* = 1.0 × 10^9^, *k_−_* = 3.0 × 10^6^ (7)

HBrO^•−^ + Cl^−^ ⇄ BrCl^•−^ + OH^−^*k_+_* = 1.9 × 10^8^, *k_−_* = 2.0 × 10^7^ (8)

As discussed above, Cl^•^ hardly existed in the reaction system and most of the HClO^•−^ was prone to reform ^•^OH and Cl^−^, so Equation (5) and Equation (7) could be neglected. Because of the close *k_+_* and *k_−_* of Equation (8), formation of BrCl^•−^ through Equation (8) was not the major path. Focusing on Equation (6), it could be seen that Br^•^ was consumed to generate BrCl^•−^, hence the formation of CH_3_Br decreased. Although no data is available for reactions involving BrCl^•−^, it is typically assumed to react with rate constants intermediate between or similar to single-halogen radicals [37,41]. Consequently, BrCl^•−^ would react with ^•^CH_3_ forming CH_3_Br or CH_3_Cl (Equation (9)). Different with chlorine radical that was hardly generated, BrCl^•−^ could be formed in the coexistence of Fe(III), Br^−^ and Cl^−^; therefore, CH_3_Cl was produced.

BrCl^•−^ + ^•^CH_3_ → CH_3_Br + Cl^−^ or CH_3_Cl + Br^−^ (9)

Halide oxidation by ^•^OH has long been recognized as a source of halogen radicals in seawater [42,43]. The concentration of Br_2_^•−^ and BrCl^•−^ may exceed ^•^OH concentrations by ~3–4 orders of magnitude. Although Cl^−^ occurs at 670-fold higher concentrations than Br^−^, Br^−^ oxidation by ^•^OH drives halogen radical production [43]. Even, Br_2_^•−^ concentration may exceed those of BrCl^•−^ by ~2.5-fold, since Br_2_^•−^ arises from further reactions of BrCl^•−^ with Br^−^ [37,41]. Consequently, bromide oxidation by ^•^OH and the further production of bromine radicals play an important role for the generation of CH_3_Br. In addition, based on results in this paper, rates of Br converting to CH_3_Br ranging from 10^−9^ (in the coexistence of Br^−^ and Cl^−^) to 10^−7^ (in the presence of Fe(III)) are equivalent to the level of biological conversion rates (10^−7^ for Schizochytrium sp., 10^−9^ for Ulkenia amoeboidea, and 10^−8^ for Aurantiochytrium sp. and Phaeocystis globose) [44,45]. Therefore, photochemical formation of CH_3_Br may partly account for the generation of CH_3_Br in the marine environmental matrix.

## 4. Conclusions

The photochemical formation of CH_3_Br from SA in aqueous bromide solutions indicates a potential natural source of CH_3_Br in the bromide-enriched environmental matrix. The inhibiting effect of chloride on the formation of CH_3_Br in the absence of Fe(III) and the simultaneous generation of CH_3_Cl from SA demonstrates the competition of Cl^−^ and Br^−^ with SA to form CH_3_X (X = Cl, Br), which also suggests that CH_3_Br was generated via the nucleophilic substitution reaction. The different effects of Fe(III) on the formation of CH_3_Br and CH_3_Cl illustrate an alternative path for CH_3_Br formation, i.e., combination of ^•^CH_3_ and ^•^Br. These results suggest that there are two formation pathways for CH_3_Br from SA, i.e., nucleophilic substitution and radical recombination, which may be in concurrence in the natural environment. This study provides an insight into the pathways of CH_3_Br formation in an aquatic environment. 

## Supplementary Materials

The following are available online at https://www.mdpi.com/1660-4601/17/6/2081/s1, Figure S1: Schematic of the device used for irradiation, Figure S2: Temperature change of the reaction solution during irradiation, Figure S3: Photodegradation of SA in aqueous bromide solutions in the presence or absence of Fe(III), Figure S4: Photolysis intermediates of SA analyzed by LC-ESI(−)-MS, Figure S5: Photolysis intermediates of SA in the presence of Fe(III) analyzed by LC-ESI(−)-MS, Figure S6: Photolysis intermediates of SA in the presence of Fe(III) analyzed by LC-ESI(+)-MS.
