# Supplementary material for: Insight Into the Formation Paths of Methyl Bromide From Syringic Acid in Aqueous Bromide Solutions Under Simulated Sunlight Irradiation
Source: Int J Environ Res Public Health. 2020 Mar 20;17(6):2081. doi: 10.3390/ijerph17062081 (PMC7142905; doi:10.3390/ijerph17062081)
Supplement: Supplementary file 1 [file ijerph-17-02081-s001.pdf]

Supplementary Material

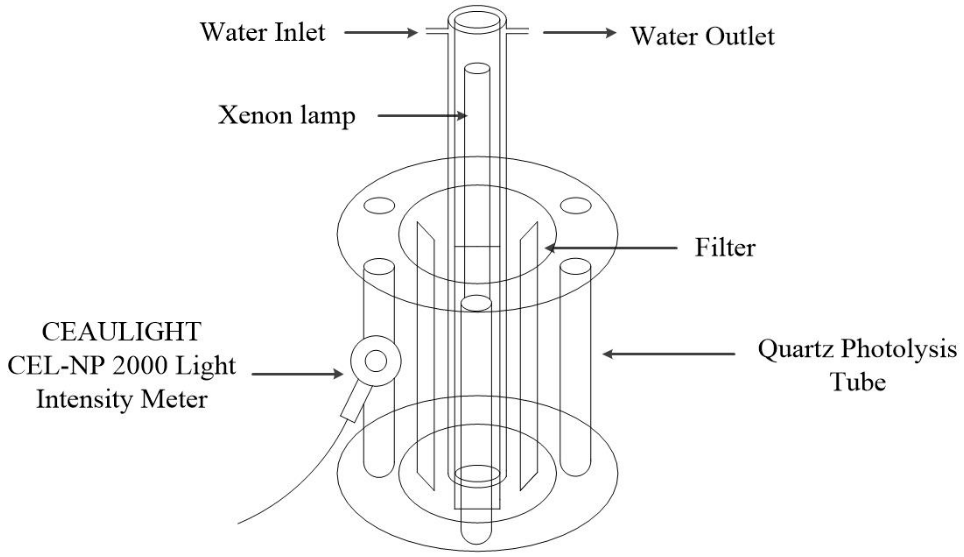

Figure S1. Schematic of the device used for irradiation.

Text S1. It is a so-called merry-go-round photo-reactor. Xenon lamp was placed in a quartz jacket where cold water (10 °C) was driven by a cooling circulating pump to take away the heat of the infrared radiation. Also, the irradiation device and the quartz tubes were cooled by a fan during irradiation. Eight pieces of filters were placed around the jacket to cut off the light with the wavelength below 290 nm. A light intensity meter (CEAULIGHT CEL-NP 2000) was used to measure the light intensity at the position where to place the quartz tube.

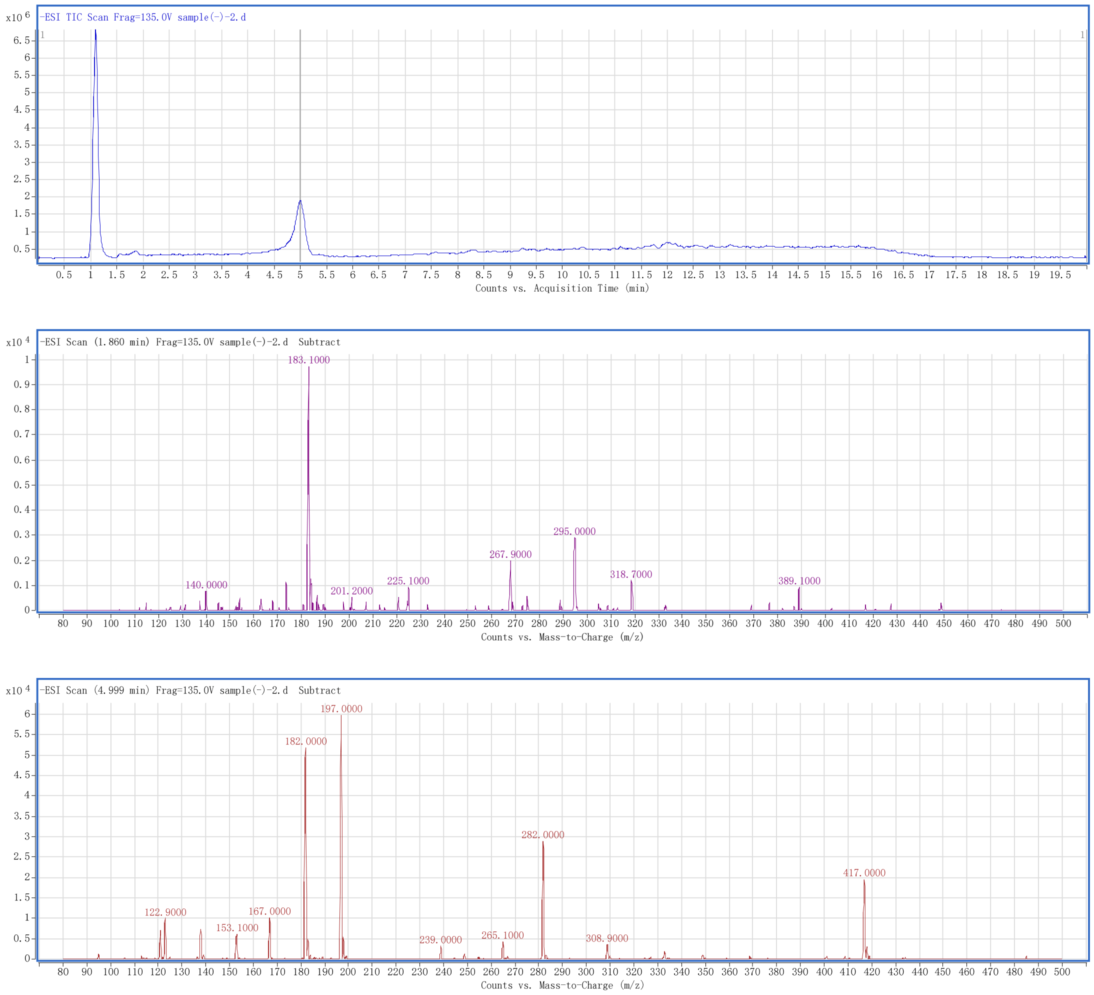

Figure S2. Photolysis intermediates of SA analyzed by LC-ESI(-)-MS, the TCI chromatogram and the MS spectra for 3-methoxy-4,5-dihydroxybenzoic acid (1.86min) and SA (4.99min).

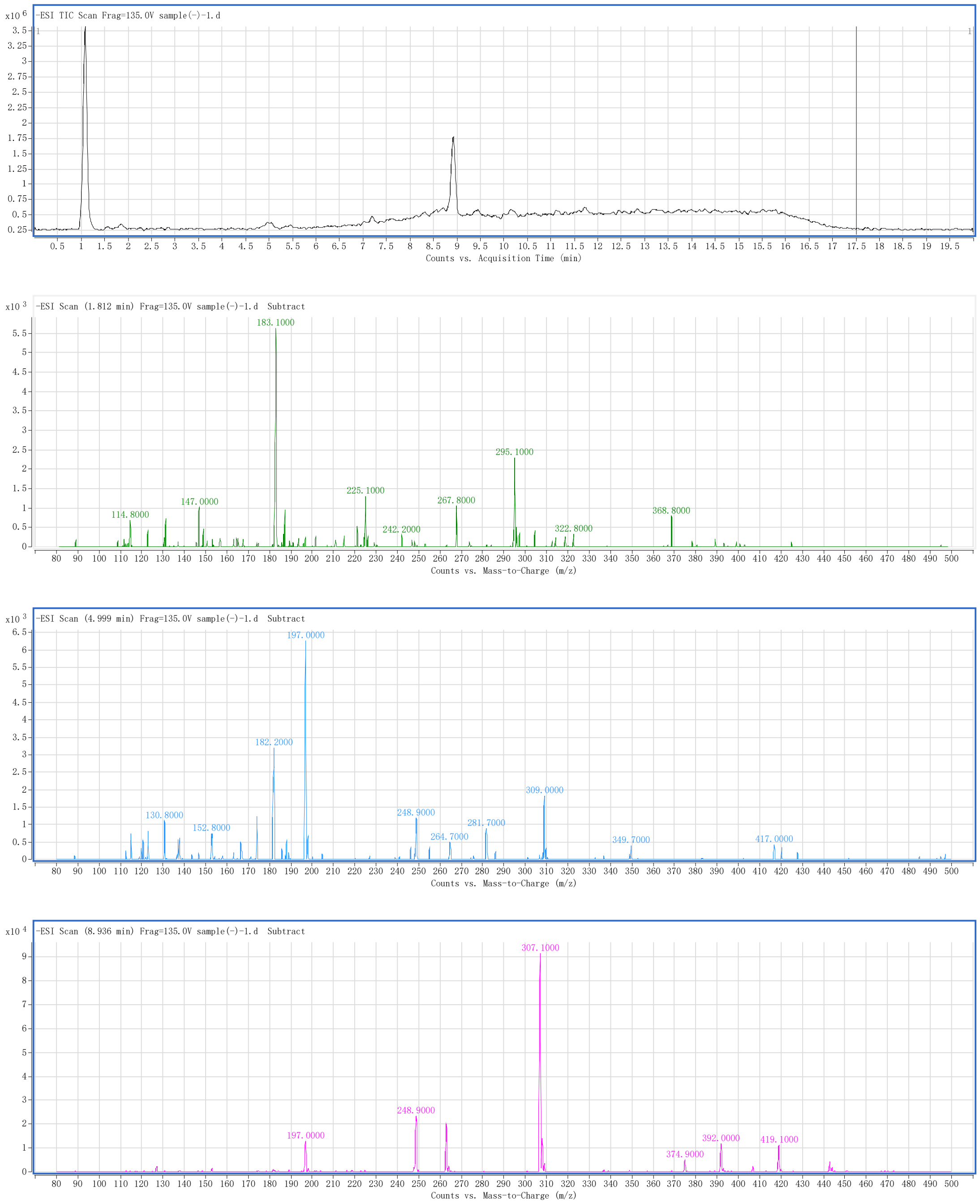

Figure S3. Photolysis intermediates of SA in the presence of Fe(III) analyzed by LC-ESI(-)-MS, the TCI chromatogram and the MS spectra for 3-methoxy-4,5-dihydroxybenzoic acid (1.81 min), SA (4.99 min) and the dimer product with MW308 (8.94 min).

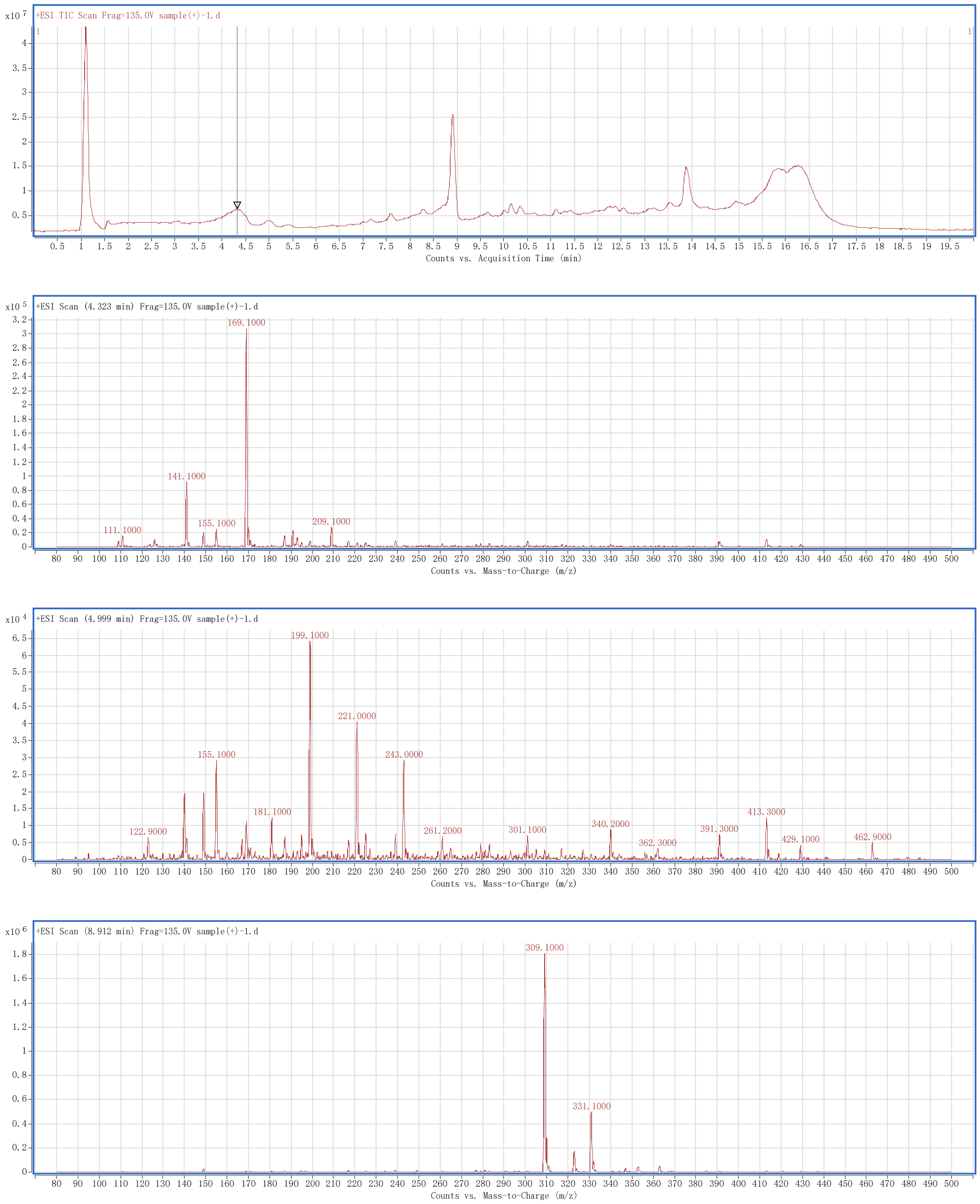

Figure S4. Photolysis intermediates of SA in the presence of Fe(III) analyzed by LC-ESI(+)-MS, the TCI chromatogram and the MS spectra for 3,5-dimethoxy-1,4-benzoquinone (4.32 min), SA (4.99 min) and the dimer product with MW308 (8.91 min). .
